# Lessons learnt from developing an ethnically diverse patient and public involvement group for breast cancer research

**DOI:** 10.1136/bmjopen-2024-091888

**Published:** 2025-03-03

**Authors:** Toral Gathani, Bep Dhaliwal, Julliet Lwiindi, Shoba Dawson

**Affiliations:** 1 Cancer Epidemiology Unit, Nuffield Department of Population Health, University of Oxford, Oxford, UK; 2 Oxford University Hospitals NHS Foundation Trust, Oxford, UK; 3 Patient representative, Oxford, UK; 4 Oxford Cancer Centre, University of Oxford, Oxford, UK; 5 Sheffield Centre for Health and Related Research, University of Sheffield, Sheffield, UK

**Keywords:** Patient Participation, Breast tumours, Health Equity

## Abstract

**Abstract:**

**Objective:**

There is evidence that those who typically contribute to patient and public involvement (PPI) activities do not reflect the diversity of the population, and individuals from underserved groups are less likely to participate in healthcare research. For some researchers, understanding how to embed diversity into their PPI work can be confusing and challenging. The aim of this communication article is to reflect on our experiences and share the lessons learnt from developing an ethnically diverse PPI group to co-deliver breast cancer research.

**Key points:**

Researchers must be realistic about timelines at both the grant application stage and during the research project, as finding contributors for inclusive and diverse PPI work takes time. Researchers will benefit from utilisation of existing expertise and resources within existing PPI teams at research institutions. It is vitally important to be clear about what researchers need in terms of contributors and what the PPI activities will be at different stages of the research project.

**Conclusions:**

Conducting effective, diverse and meaningful PPI is a research skill that needs to be learnt and practised just like any other. Well-developed inclusive PPI has significant benefits for both researchers and the public.

## Introduction

 Patient and public involvement (PPI) activities are recognised as an integral part of the research lifecycle, from developing the research question and obtaining funding to disseminating the key findings. Underserved groups are generally under-represented in healthcare research and PPI activities, which impacts the generalisability of the study findings.^1^,^3^ Additionally, there is evidence that those who typically contribute to PPI activities do not reflect the diversity of the wider population with respect to characteristics such as age, sex and ethnicity as well as others.^4^

All the major research councils and charitable funders have signed up to the UK Research and Innovation ‘Shared commitment to improve public involvement in research’.^5^ At the heart of this commitment is a requirement for inclusive PPI activity to be embedded throughout, including at the grant application stage.^6^ Similarly, the Royal Society of Chemistry has brought together 56 publishing organisations to set a new standard to ensure a more inclusive and diverse culture within scholarly publishing.^7^ The National Institute for Health and Care Research (NIHR) has recently made inclusive research design a condition of funding.^8^

There are several resources available to help researchers deliver inclusive research, such as the National Institute for Health and Care Research INCLUDE framework,^3 9^ and the guidance developed by the Trial Forge^10^ and Step Up^11^ groups. There is also guidance available from several sources on how to develop and deliver PPI^12^ and what the expected standards are.^13^ However, recruiting inclusive PPI groups can be challenging. Recognised barriers to engaging with PPI include having time and finance to participate and an awareness of opportunities. These factors may disproportionately affect potential contributors from diverse backgrounds.^14^

Researchers at all stages of their careers can find delivering inclusive and meaningful PPI as part of their research confusing and challenging.^15^ Our research is focused on ethnicity and breast cancer, and in this communication article, we have reflected on our own experiences of setting up an ethnically diverse PPI group to work with us to deliver our research. Our aim is to share the steps we took and the lessons we learnt to provide support and guidance to other researchers.

## Our research project

The aim of our research project is to investigate the associations between ethnicity and breast cancer outcomes. The work is funded by Cancer Research UK and will run for at least 3 years.^16^ The research team is based in the Cancer Epidemiology Unit at the Nuffield Department of Population Health at the University of Oxford.

Breast cancer is the most commonly diagnosed cancer in women of all ethnic groups in the UK.^17^ Data from the most recent Office for National Statistics 2021 census shows that the largest single ethnic groups in the UK are white, Indian, Pakistani, Black African and Black Caribbean.^18^ In the UK, ethnicity is self-reported using predefined categories that reflect the ethnic population in the national census and health records.^19 20^ Previous studies have shown that although women from ethnic minority groups are less likely to be diagnosed with breast cancer compared with white women,^21^ they are more likely to be diagnosed with a more advanced disease at presentation, which is associated with poorer outcomes.^22 23^

During the grant development stage, we consulted with members of existing local Public Advisory Panels within our research department on the development of the research aims, the PPI plan and the lay summary for the study. These contributors were a mixture of healthy women and breast cancer survivors and included contributors from ethnic minority groups. We also ensured that sufficient funding was requested to be able to convene a more permanent ethnically diverse PPI group to deliver the PPI activities needed over the duration of the research project. Researchers need to plan the PPI activities that are needed for a grant application and build in sufficient lead time so that this element is not rushed.

Our research aims to build on previous work and broadly are to:

1. Describe the incidence rates and risk factor profiles for breast cancer in women from five specific ethnic groups: white, Indian, Pakistani, Black African and Black Caribbean.
2. Evaluate the barriers and enablers for the early diagnosis of breast cancer among women from these ethnic minority backgrounds.
3. Investigate the differences in the tumour characteristics of breast cancer diagnosed in women from different backgrounds, taking into account known risk factors.

The first and second research aims involve desk-based projects using existing data. For these aims, the PPI activities will largely involve dissemination of the research findings. We envisaged that these activities would include the co-creation of newsletters, lay summaries, infographics, animations and video clips to share the study findings through relevant community networks and communication channels, using traditional approaches and social media.

The third research aim will require the generation of new data through a future hospital-based study, and for this aim, PPI activities will be embedded throughout the research lifecycle and will include co-creation of lay study material, activating and engaging with community networks and contribution to publicising the study, and providing advice on the management of potential digital and literacy and other relevant barriers to participation, as well as the PPI activities for dissemination as described above.

## The Ethnicity and Breast Cancer PPI group

To support delivery of the research aims and associated PPI activities, we planned to convene a new ethnically diverse PPI panel from a broad range of backgrounds with ~15–20 contributors. The inclusion criteria were either women without a history of breast cancer or women with a lived experience of breast cancer, and to be from one of our five ethnic groups of interest (white, Indian, Pakistani, Black African and Black Caribbean). We planned to recruit four members from each of our five ethnic groups of interest and to include at least one woman without a history of breast cancer and one woman with a lived experience of breast cancer. The planned oversampling of the ethnic minority groups was deliberate and appropriate given the overall study aims. The arrangement allowed for flexibility, for both the study team and PPI contributors, to constitute different types of panels depending on the involvement activity planned at different stages of the research as well as enabling the contributors to also choose the extent of their involvement.

The process of setting up the group took place in three phases which were planning, recruitment and ongoing management:

### Planning

The research project team partnered with the PPI team at the Oxford Cancer Centre (also funded by Cancer Research UK) to assist with the recruitment and management of the Ethnicity and Breast Cancer PPI group. There was a natural synergy to the partnership as the Oxford Cancer Centre PPI team was keen to support PPI for research in underserved populations and to work with researchers to reduce the barriers to participating in PPI for these groups.

By partnering with an established team that manages several PPI groups, we avoided duplication of effort and were able to adapt existing resources. This ensured that the governance of our PPI group was similar to others, and as such, all PPI contributors were being treated similarly. For example, we adapted existing Oxford Cancer PPI terms of reference to include involvement fees for this particular group. There is variation among funders for the recommended involvement fees for PPI activity. Therefore, it is important to provide clarity about fees and what other expenses will be covered, for example, travel, childcare, etc in all communications with potential contributors to manage expectations and ensure transparency.

Particular benefits of working in this type of synergistic partnership included:

- Leveraging existing knowledge of and relationships with community organisers and groups.
- Providing the PPI contributors with the opportunity to participate in the wider Oxford Cancer Centre PPI group and have access to a range of online and in-person events and be provided with training and ongoing support.

- Developing a collaboration within the University of Oxford which facilitates ongoing opportunities for shared learning among researchers from different groups and disciplines.

### Recruitment

Recruiting to a new group, especially in communities that are not necessarily aware of different PPI opportunities or are generally under-represented in research, took significantly longer than we expected. The opportunity to join a PPI group has to be widely advertised, and consideration has to be given to how to target particular communities. All communications need to be written clearly in lay language and with avoidance of scientific and/or technical terms. Our recruitment process has involved repeated targeted advertising, followed by an initial screening of potential contributors and a subsequent meeting between contributors and study investigators. Research teams are encouraged to have realistic expectations of how long this process can take and plan timelines accordingly. This process of advertising, screening and holding a first meeting can take around 8–12 weeks for each wave of recruitment.

#### Advertising

We leveraged multiple channels including online platforms, local and community-specific newsletters and email groups. We compiled a list of organisations and groups to contact, and used a snowball approach to find further contacts to approach. All initial contact was made online. These organisations included breast cancer charities, for example, Breast Cancer Now; patient advocacy groups, for example, Black Women Rising; existing PPI groups, for example, the Oxford Cancer Centre PPI group and community-based organisations particular to our ethnic groups of interest, for example, The Leanne Pero Foundation.

**Figure 1:**
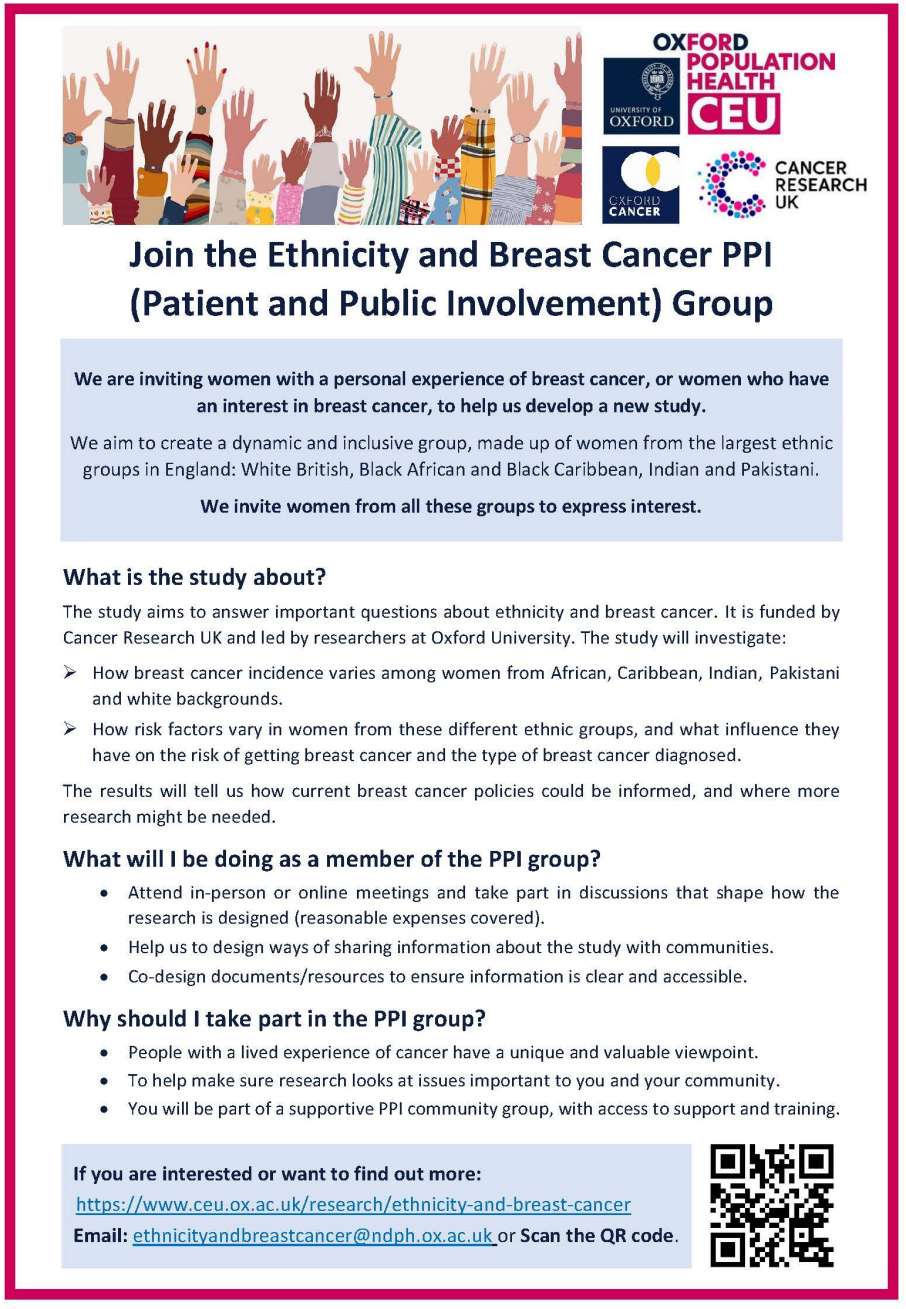
Example of recruitment flyer for Ethnicity and Breast Cancer PPI group.

We designed a recruitment flyer (figure 1) containing brief details and links to the study website to share through their mailing lists and social media accounts. We were keen to ensure that our flyer was relevant and reflected the diversity of the wider population and the individuals that are the main focus of this recruitment. The images selected included people from a variety of backgrounds and ethnicities, and aimed to provide a personalised approach to these women based on their lived experience.

Advertising the opportunity for participation in the PPI group was an iterative process, and at the time of writing, we have conducted four waves of advertising, targeting different organisations and communities, or re-advertising with some of them depending on the recruitment needs each time.

#### Screening of potential contributors

Potential contributors express interest in participation by completing a short user-friendly online registration form (https://www.cancer.ox.ac.uk/support/PPI/interest-form) or sending an email to a generic Oxford Cancer PPI team address that is regularly monitored. The expressions of interest were screened against the criteria for inclusion in the PPI group, and potential contributors were offered an initial one-to-one 30 min meeting online to explain the study, the role of the PPI group and explore reasons for participation.

The main purpose of these initial meetings was to establish if the needs, expectations and experiences of the contributors are aligned with the needs of the PPI group. It was our experience that some potential contributors were seeking breast cancer support groups or patient advocacy groups and were redirected to appropriate resources at that point. This meeting also provided an opportunity to start an initial conversation with potential contributors about their introduction to the concept of PPI activities and what it entails and how their involvement would benefit the project and how their involvement and contributions could benefit their own communities.

#### Meeting with study investigators

Following the initial meeting, potential contributors were invited to attend a larger group meeting at which the study investigators were present. Potential contributors were asked about their preferred location for this meeting (in-person or virtual). The potential contributors were informed that these initial in-person meetings would be held in a meeting room in the research department and no concerns about location were raised.

We have conducted three in-person meetings and one online meeting. Our experience is that in-person meetings allow for a deeper and richer set of interactions between attendees and provide an opportunity for both formal and informal conversations to take place. As such, we would recommend that ideally the initial meeting between PPI contributors and study investigators should take place in person. The timing of the meeting should consider the potential needs of the contributors, and care should be taken to avoid significant religious events and school holidays. All travel costs and expenses related to attending an in-person meeting were covered by the research team. Involvement fees were provided to all contributors who attended a meeting in person or online.

The purpose of the meeting was for the study team and potential contributors to meet and to evaluate whether participation in the PPI group is right for both parties. It was made clear at the time of invitation that attending this meeting did not commit a contributor to ongoing involvement. Similarly, attending the meeting did not mean that an offer to join the PPI group would definitely follow.

The format of each meeting was similar with a presentation from the PPI team to explain broadly what PPI is and how it works, followed by a presentation from a study investigator about the research and what the PPI will involve. After the presentations, we had a round table discussion with refreshments where each contributor was invited to share their journey and motivation for joining the PPI group, and any questions about the study and/or PPI activities were answered.

Following the meeting, all contributors were contacted by the PPI team to thank them for their time and contribution and to arrange remuneration of fees and expenses. We invited all contributors to be part of the wider PPI group for the Oxford Cancer Centre. Suitable contributors were formally invited to join the Ethnicity and Breast Cancer PPI group, and we have invited one contributor to join the study team. Currently, we have 14 active PPI members who represent all our ethnic groups of interest, but we continue to engage with organisations and groups to identify further potential contributors.

### Ongoing management

Following initial meetings, ongoing communication with PPI contributors has to be maintained to help ensure that they feel valued, supported and are part of the research project. All members of our group were provided with a named point of contact for any ongoing queries.

Regular email contact is maintained through general communications from the wider Oxford Cancer PPI team about events and training opportunities, as well as a specific Ethnicity and Breast Cancer newsletter twice a year.

Moving forwards, we will bring together all members of the Ethnicity and Breast Cancer PPI group for at least one in-person meeting annually as an opportunity to provide contributors with an update on study progress, as well as to have general discussions about PPI. At other times, depending on the type of PPI activity needed, we will hold meetings either physically or virtually, and/or seek input via email communication.

## Reflections of our experience

Overall, our experience of establishing a dedicated Ethnicity and Breast Cancer PPI group has been positive, and the same principles of managing and delivering PPI groups apply. On reflection, the most important lessons we learnt are:

- Finding contributors for inclusive and diverse PPI takes more time than anticipated. Realistic timelines are needed at the grant application stage and during the research project if funding is awarded. Effort must be made to identify and engage with a range of other stakeholders to facilitate reaching different groups of potential contributors.
- There is a vast range of existing expertise and resources available to researchers, and early engagement with the PPI teams at your institution will benefit your research. Partnering with established teams who manage PPI groups is essential to avoid duplication of efforts by harnessing resources and to ensure equity of experience for contributors.
- It is vitally important to be clear and specific about what you are looking for in terms of contributors and what the planned PPI activities will be at different stages of the research project.
- Well delivered PPI is an opportunity for shared learning which will enhance the understanding and reach of your own research.

We asked our patient contributor and co-author to share her thoughts on why contributing to this PPI group was important to her.

‘I joined the Ethnicity and Breast Cancer Public and Patient Involvement group to share my voice and lived experience of my breast cancer diagnosis at the age of 41 years. At the time I was diagnosed, I had not met anyone else who looked like me and had also experienced a cancer diagnosis, and I was not emotionally prepared for the shame and stigma that came with it. The treatments for my cancer, which included surgery, chemotherapy and radiotherapy, were physically and emotionally challenging. I did not have anyone who could really understand the impact my cancer diagnosis has had on the roles I played in my family and in my community, as well as on myself.

This experience has led me to be active in PPI and community engagement to try and ensure that others who look like me do not feel isolated and alone while navigating a life-changing diagnosis. I want to support research and awareness of breast cancer in minority communities so that people talk more openly in families, and collectively we address fear and stigma which ultimately could save lives.’

## Conclusion

Conducting effective, diverse and meaningful PPI is a research skill that needs to be learnt and practised just like any other. Researchers, at all stages of their career, should attend courses and familiarise themselves with key principles and standards of conducting PPI and seek to incorporate PPI at the earliest stage of their research projects and proposals. Well-developed inclusive PPI has significant benefits for both researchers and the public.
