# Supplementary material for: Lessons learnt from developing an ethnically diverse patient and public involvement group for breast cancer research
Source: BMJ Open. 2025 Mar 3;15(3):e091888. doi: 10.1136/bmjopen-2024-091888 (PMC11877151; doi:10.1136/bmjopen-2024-091888)
Supplement: Supplementary file 1 [file bmjopen-2024-091888.reviewer_comments.pdf]

PEER REVIEW HISTORY

BMJ Open publishes all reviews undertaken for accepted manuscripts. Reviewers are asked to complete a checklist review form and are provided with free text boxes to elaborate on their assessment. These free text comments are reproduced below.

ARTICLE DETAILS

Title (Provisional)

Lessons learnt from developing an ethnically diverse Patient and Public Involvement group for breast cancer research

Authors

Gathani, Toral; Dhaliwal, Bep; Lwiindi, Julliet; Dawson, Shoba

---

VERSION 1 - REVIEW

**Reviewer**	**1**
**Name**	**Martei, Yehoda**
**Affiliation**	**University of Pennsylvania Perelman School of Medicine, Medicine (Hematology-Oncology)**
**Date**	**15-Jan-2025**
**COI**	**None**

The manuscript by Gethani et al addresses the important issue of diverse representation in PPI for breast cancer research and share lessons from their experience. Overall, although this is an important subject, there are several details that are missing, and in its current format, doesn't offer in-depth guidance for PPI group formation.

My comments are summarized below by sections:

Our research project:

Page 2, line 42, because of differences in the definition of ethnicity – the authors should specify how these are defined in the UK, is this self-identified ethnicity?

Page 3, line 12 – the authors emphasize the need to include PPI activities throughout the research conceptualization to dissemination process, yet for their study, they only included PPI activities for the dissemination process – can the authors comment on their decision to involve PPI groups at the end of their study for dissemination purposes?

Ethnicity and breast cancer PPI group

Page 3, line 27-28, Oversampling of minority groups in their PPI composition is appropriate given the study aims.

Recruitment

Page 4, line 15-16, can the authors provide more data on how long the process took to provide a reference frame for future researchers for allocating time for PPI recruitment?

Advertising:

Page 4, 33-35: The authors should provide examples of organizational groups contacted and modality of advertising, were there in-person outreach? What did the 4 waves focus on during the advertising period? Was there consideration for non-English speakers and translation to other languages? Similar to above, can you provide an estimate of time involvement.

Meeting with study investigators:

Page 4, line 53-54: A bit more nuanced, but in working with PPIs, leaders in community engagement have advocated for in-person meetings that occur within the community, ie outside of the academic institution, creating a safe space and equity in the partnership. The authors mention some in-person meetings without providing details on actual location of these meetings which is important.

Page 5, line 3-5 – Excellent emphasis on paying PPI group members and reimbursing travel costs. The authors should provide data on the final composition for the PPI, was there purposive sampling to also ensure not just ethnic diversity but socioeconomic diversity as well?

Reflections of our experience:

As highlighted above in other areas – data should be presented on actual time it took, why was the PPI group not engaged earlier in the conceptualization and design of the study and finally comment on whether ethnic diversity was achieved.

Page 6, line 16 – the statement from the PPI member highlights one of her goals, to address fear and stigma, does any part of your study address this? How do you ensure alignment with PPI member goals or consider that in your research?

**Reviewer**	**2**
**Name**	**Griffiths, Sarah**
**Affiliation**	**UCL, Primary Care and Population Health**
**Date**	**20-Jan-2025**
**COI**	**None**

The article reports on the important topic of increasing diversity within public involvement and has potential to inform the reader on valuable strategies to achieve this. However the

strategies described in the article largely apply to PPI recruitment and retention in general rather than to recruiting people from under served groups. This is a missed opportunity to share some nuggets of advice relating to how to engage with and involve the minority ethnic groups described. I would encourage the authors to revisit this paper and include more in depth reflections to address this.

Specific comments:

As well as citing policy and guidelines, reference should be made to research already published on recruiting diverse populations to PPI work.

It is unclear why the Royal Society of chemistry has been referenced as opposed to other scholarly societies.

In the introduction it is not clear what is meant by diversity. It should be made clearer that the focus of the paper is on ethnicity, unless diversity is also relevant in terms of other characteristics e.g., age, sexuality, socioeconomic status.

Line 51 could be more specific in terms of which ethnic minority groups (or minority ethnic groups)

line 58 first research aim - again be specific about which specific groups.

Page 2 line 29 - what are the 5 ethnic groups? It's not obvious to the reader until reading the flyer

page 2 line 51 - consider the wording e.g., clarity about the/regarding the funding? This also is an example of a strategy that support recruitment generally in PPI.

section on screening - examples of screening questions would be helpful. Was the issue of payment for the initial 30 minute screening meeting considered?

Holding the first meeting in person is justified but how did the contributors feel about it? some people find it hard to travel for instance.

General points:

What were the results in terms of how many people from each targeted group were recruited to be involved?

Given the objective to share reflections on embedding diversity within PPI, what's missing are reflections on specific barriers and facilitators to engaging people from the different minority groups, rather than general reflections on factors that support engagement of all groups.

**Reviewer**	**3**
**Name**	**Rattray, Zahra**

Affiliation **University of Strathclyde, Strathclyde institute of pharmacy and biomedical sciences**

Date **24-Jan-2025**

COI **None**

The authors share their reflections and experiences on inclusive PPI in healthcare. The topic of this publication is timely and would be beneficial to researchers wanting to engage in meaningful PPI. Developing a framework in this context would be beneficial.

To make the publication more useful to implement by the broader researcher community, I recommend the following revisions to be made:

In the introduction, there is a lack of context on the systemic barriers to minority ethnic participation in PPI.

Sharing of the resources such as the registration forms would benefit the readership

P4- In the design of PPI activities, what numbers of patients and costing elements were considered- a concise indication of this would be beneficial.

P4/5- some of the text is framed in future/present prose, while some elements are presented in past prose- I recommend that consistency is adopted in framing this

A brief account of limitations/areas that are needed to facilitate inclusion are needed

---

VERSION 1 - AUTHOR RESPONSE

Reviewer: 1 Dr. Yehoda Martei, University of Pennsylvania Perelman School of Medicine

Comments to the Author:
The manuscript by Gathani et al addresses the important issue of diverse representation in PPI for breast cancer research and share lessons from their experience. Overall, although this is an important subject, there are several details that are missing, and in its current format, doesn't offer in-depth guidance for PPI group formation.

My comments are summarized below by sections:
Our research project:
Page 2, line 42, because of differences in the definition of ethnicity – the authors should specify how these are defined in the UK, is this self-identified ethnicity?
Authors' response: Thank you for this comment. A sentence and reference has been added to Page 2 Line 35 explaining recording of ethnicity in health records and census.

Page 3, line 12 – the authors emphasize the need to include PPI activities throughout the research conceptualization to dissemination process, yet for their study, they only included PPI activities for the dissemination process – can the authors comment on their decision to involve PPI groups at the end of their study for dissemination purposes?
Authors' response: Thank you for this comment. We have reworded lines 5-18 to make it clearer that PPI activities are included throughout the research lifecycle for the third aim which involves the generation of new data. For the first and second aims, which use existing data that has

already been collected, the main PPI activities are for dissemination.

Ethnicity and breast cancer PPI group:
Page 3, line 27-28, Oversampling of minority groups in their PPI composition is appropriate given the study aims.
Authors' response: Thank you for this comment. We have added a sentence Page 3 Line 31

Recruitment:
Page 4, line 15-16, can the authors provide more data on how long the process took to provide a reference frame for future researchers for allocating time for PPI recruitment?
Authors' response: Thank you for this comment. A sentence has been added to Page 4 Line 25

Advertising:
Page 4, 33-35: The authors should provide examples of organizational groups contacted and modality of advertising, were there in-person outreach? What did the 4 waves focus on during the advertising period? Was there consideration for non-English speakers and translation to other languages? Similar to above, can you provide an estimate of time involvement.
Authors' response: Thank you for this comment. Details have been added Page 4 lines 29-34.

Meeting with study investigators:
Page 4, line 53-54: A bit more nuanced, but in working with PPIs, leaders in community engagement have advocated for in-person meetings that occur within the community, ie outside of the academic institution, creating a safe space and equity in the partnership. The authors mention some in-person meetings without providing details on actual location of these meetings which is important.
Authors' response: Thank you for this comment. We have added a sentence to Page 5 line 18.

Page 5, line 3-5 – Excellent emphasis on paying PPI group members and reimbursing travel costs. The authors should provide data on the final composition for the PPI, was there purposive sampling to also ensure not just ethnic diversity but socioeconomic diversity as well?
Authors' response: Thank you for this comment. We did not conduct purposive sampling for socioeconomic diversity at this time but will consider ways to incorporate this moving forward. A sentence has been added Page 5 Line 42.

Reflections of our experience:
As highlighted above in other areas – data should be presented on actual time it took, why was the PPI group not engaged earlier in the conceptualization and design of the study and finally comment on whether ethnic diversity was achieved.
Authors' response: Thank you for this comment. Our research is ongoing and developing the PPI group is one of our first steps, and a sentence has been added to Page 3 Line 1 to clarify. PPI members were consulted on the research proposal and engaged with from the conceptualisation of the study as outlined Page 2 line 41.

Page 6, line 16 – the statement from the PPI member highlights one of her goals, to address fear and stigma, does any part of your study address this? How do you ensure alignment with PPI member goals or consider that in your research?
Authors' response: Thank you for this comment. One of the aims of our research is to explore the barriers and enablers to early diagnosis and fear and stigma will be explored. Our research is ongoing and the PPI member voices will be included in final outputs.

Reviewer: 2 Dr. Sarah Griffiths, UCL

Comments to the Author:
The article reports on the important topic of increasing diversity within public involvement and has potential to inform the reader on valuable strategies to achieve this. However the strategies described in the article largely apply to PPI recruitment and retention in general rather than to recruiting people from underserved groups. This is a missed opportunity to share some nuggets of advice relating to how to engage with and involve the minority ethnic groups described. I would encourage the authors to revisit this paper and include more in depth reflections to address this.

Authors' response: Thank you for this observation. Together with this suggestion and those from the other reviewers, we have made changes throughout the manuscript to provide a more in-depth discussion about including underserved groups.

Specific comments:
As well as citing policy and guidelines, reference should be made to research already published on recruiting diverse populations to PPI work.

It is unclear why the Royal Society of chemistry has been referenced as opposed to other scholarly societies.
Authors' response: Thank you for this comment. The Royal Society of Chemistry is specifically mentioned because they led on bringing together 56 publishing organisations to set standards – lines 11-13 page 2.

In the introduction it is not clear what is meant by diversity. It should be made clearer that the focus of the paper is on ethnicity, unless diversity is also relevant in terms of other characteristics e.g., age, sexuality, socioeconomic status.
Authors' response: Thank you for this comment. For clarification, detail has been added to line 7 page 1 and to lines 19-20.

Line 51 could be more specific in terms of which ethnic minority groups (or minority ethnic groups) line 58 first research aim - again be specific about which specific groups.
Authors' response: Thank you for this comment – detail added to Line 7 Page 3

Page 2 line 29 - what are the 5 ethnic groups? It's not obvious to the reader until reading the flyer
Authors' response: Thank you for this comment – detail added to Line 7 Page 3

Page 2 line 51 - consider the wording e.g., clarity about the/regarding the funding? This also is an example of a strategy that support recruitment generally in PPI.
Authors' response: Thank you for this comment – the sentence on line 1-4 page 3 has been reworded

Section on screening - examples of screening questions would be helpful. Was the issue of payment for the initial 30 minute screening meeting considered?
Authors' response: Thank you for this comment – a sentence has been modified Lines 42-43 on page 4.

Holding the first meeting in person is justified but how did the contributors feel about it? some people find it hard to travel for instance.
Author's response: Thank you for this comment – a sentence has been added to Lines 18-20 on page 5.

General points:

What were the results in terms of how many people from each targeted group were recruited to be involved?
Authors' response: Thank you for this comment. A sentence has been added Page 5 Line 42.

Given the objective to share reflections on embedding diversity within PPI, what's missing are reflections on specific barriers and facilitators to engaging people from the different minority groups, rather than general reflections on factors that support engagement of all groups.
Authors' response: We have added some thoughts to the section on Reflections.

Reviewer: 3 Dr. Zahra Rattray, University of Strathclyde
Comments to the Author:
The authors share their reflections and experiences on inclusive PPI in healthcare. The topic of this publication is timely and would be beneficial to researchers wanting to engage in meaningful PPI. Developing a framework in this context would be beneficial.
Authors' response: We thank the reviewer for this comment and will consider the steps needed to develop a formal framework for the work we have done.

To make the publication more useful to implement by the broader researcher community, I recommend the following revisions to be made:
In the introduction, there is a lack of context on the systemic barriers to minority ethnic participation in PPI.
Authors' response: Thank you for this comment. We have added a paragraph to the introduction.

Sharing of the resources such as the registration forms would benefit the readership.
Authors' response: Thank you for this comment. These forms are in the public domain and a weblink has been added to the text Line 3 Page 5

P4- In the design of PPI activities, what numbers of patients and costing elements were considered- a concise indication of this would be beneficial.
Authors' response: Thank you for this comment. All elements for the PPI activities were costed in accordance with Cancer Research UK guidance during the grant application process. These applications are reviewed by both a scientific panel and a PPI panel and approved. We make reference to the variation among funders for the recommended involvement fees in Line 5 page 4.

P4/5- some of the text is framed in future/present prose, while some elements are presented in past prose- I recommend that consistency is adopted in framing this
Authors' response: Thank you for the comment – we have gone through the text and harmonised the use of tense throughout.

A brief account of limitations/areas that are needed to facilitate inclusion are needed.
Authors' response: Thank you for this comment. We feel that we have covered a lot of these elements in section 2 on the recruitment approach.
